# Keeping track of and recognizing the value of Public Involvement work in dementia research

**DOI:** 10.3389/fneur.2022.1031831

**Published:** 2022-11-11

**Authors:** Jean Georges, Ana Diaz-Ponce, Daphne Lamirel, Soraya Moradi-Bachiller, Dianne Gove

**Affiliations:** Alzheimer Europe, Luxembourg, Luxembourg

**Keywords:** dementia, Public Involvement, research, public-private partnership, neurodegeneration

## Abstract

The Public Involvement (PI) of people with dementia is slowly but progressively moving from a “nice to have” to a “must have” element of good-quality dementia research. Research funders and ethics committees increasingly ask for evidence of the planning of such involvement. The actual conduct and outcome of PI are, however, unfortunately typically under or inadequately reported. In this article, we provide an overview of what PI is and why it is important to dementia research and Alzheimer Europe's approach to PI. We draw on our recent experience of compiling a set of examples of PI in different European projects in publicly available sources. This highlighted the difficulty of finding information about PI activities and the almost total lack of details of such activities in formal reports, official records, and/or public project websites. In this article, we emphasize gaps and call for more stringent conditions for the inclusion and reporting of PI work in the context of the approval and funding of dementia research projects. We call for the establishment of obligatory reporting on the nature, specific challenges, and impact of PI in dementia research in formal reports (e.g., to funders), in public project websites, and in peer-reviewed articles. Such reporting should cover several key factors such as who was involved, how they were involved, and what impact PI had on the research process.

## What is Public Involvement and why is it important to dementia research?

Public Involvement (PI) in the field of dementia research is about the active involvement of people with dementia in research projects other than as research participants. It may also involve people who are at risk of developing dementia, members of the general public, informal (unpaid) carers, and people who use, or have used, health and social care services concerning dementia. PI can take many different forms but typically involves members of these groups working together with researchers and sharing their perspectives, experiences, and needs with regard to the research topic, design, and conduct of the study. This differs from Public Engagement, which can be defined as raising awareness, stimulating interest, and disseminating information and knowledge to the general public (including patients) about research studies and topics. However, these two terms have developed independently in different countries and contexts. Many different terms are used such as Public Involvement (PI), Public Engagement (PE), Patient and Public Involvement (PPI), Patient, Carer and Public Involvement (PCPI), and Patient and Public Involvement and Engagement (PPIE). The lack of clarity and consistency about the terminology contributes toward confusion about the concept itself and hampers efforts to promote it as an essential part of good-quality dementia research.

Drawing on Ives et al. (1) and Gradinger et al. (2), the two main objectives of PI work in dementia research can be summarized as follows:

1. to give people with dementia a voice in research that is relevant to their lives and well-being (linked to democratic decision-making, public accountability, legitimization, and transparency, as well as the right to voice),
2. to improve the research process and outcomes, affecting the quality, relevance, and/or utility of research (both from a research and user perspective), and to provide knowledge that might otherwise be missing (e.g., highlighting issues and asking questions about things that researchers have perhaps not considered, often drawing on personal experience within a non-medical or technical frame of reference (3).

The involvement of people with dementia throughout the whole process of research (starting with the identification of the topic through to the dissemination of the results to lay audiences) helps researchers to develop methods and tools that are best suited to participants' needs (potentially improving recruitment, retention, and compliance) and ensuring that research is also meaningful in the sense of addressing worthwhile topics for people with dementia and society as a whole.

In this article, we describe our approach to PI and briefly reflect on a recent experience of identifying examples of PI in European projects in the field of neurodegenerative research, based on publicly available information. We emphasize gaps and call for more stringent conditions for the inclusion and reporting of PI work in the context of the approval and funding of dementia research projects.

## What is AE's approach to Public Involvement?

AE has always been keen to promote the involvement of people with dementia in its work and, more specifically, in dementia research. The involvement of people with dementia started several years ago in a more ad hoc manner, but has been consolidated and expanded over the years: This was done through the setting up of the European Working Group of People with Dementia (EWGPWD) in 2012 and, more recently, the development of some project-linked Advisory Boards also involving people at risk of developing dementia.

Over the years, the organization has adopted an inclusive person-centered approach to PI in research. Several aspects of this approach have been described in different academic papers including a Position Paper on Public Involvement in dementia research (written in collaboration with members of the international network of psychosocial researchers INTERDEM and people with dementia from the EWGPWD) and a report on inclusive research (4). With regard to terminology surrounding PI, whilst we used the term PPI in our earlier work, we replaced this with the term PI following discussions with people with dementia at Alzheimer Europe's annual conference in 2020. This was in response to objections from members of national dementia working groups, as well as from members of the EWGPWD, to being labeled and positioned as patients outside of their specific interpersonal doctor-patient relationships.

Relevant elements of this approach include:

• Ensuring that the PI activities are carefully planned and are timely, meaningful, and correspond to individual interests, wishes, and abilities.
• Thinking in terms of diversity (instead of representation), which involves listening to the perspectives and learning from the lived experience of very different people with dementia.
• Providing the necessary support for the people involved to be able to meaningfully and confidently participate in the PI activities, including, for example, providing accessible information in advance of the meeting about the topic to be addressed and facilitating the meeting in a manner that promotes the meaningful participation of everyone involved.
• Building and maintaining mutually respectful relationships between people with dementia and researchers, which also includes acknowledging the work of the people with dementia involved and providing feedback about the way their input has (or has not) been used and its impact on the research.

This approach is, however, not set in stone and continues to change and evolve. AE has been responsible for the PI activities of several European-funded research projects, many of which have been supported by the Innovative Medicines Initiative (IMI), which is a public-private partnership (PPP) between the European Union (European Commission) and the European pharmaceutical industry (EFPIA, the European Federation of Pharmaceutical Industries and Associations). AE has been a full partner in several IMI-funded projects, including among others the “Real world Outcomes across the Alzheimer's Disease spectrum for better care: Multi-modal data Access Platform (ROADMAP)” and the “Remote Assessment of Disease and Relapse—Alzheimer's Disease” (RADAR-AD) projects. In the ROADMAP project, which was conducted between 2016 and 2018, we involved people with dementia in a one-off activity that had a significant impact on the project as it was about the conceptualization of the progression and staging of dementia, their views on what constitutes a meaningful delay of the disease, and their feedback on a European survey for people with dementia and carers.

More recently, in RADAR-AD, a project-specific Patient Advisory Board was set up and has been providing feedback from the beginning of the project to all work packages involved. This work has shown the benefits and challenges of bringing together people affected by dementia, researchers, and representatives from the pharmaceutical industry in the context of research. For example, working collaboratively in this way with several stakeholders and different companies may be easier for people affected by dementia than working with one single company (e.g., in terms of trust, timing, confidentiality issues, etc.). Details of the PI activities carried out within these projects have been published elsewhere (5–7).

## What are the gaps with regard to PI in dementia research?

AE is not the only organization working in this way in Europe. In many, but not yet all, countries, PI in dementia research has been gradually growing over the last decade. Some European funding programmes, such as the Joint Programme for Neurodegenerative Diseases (JPND) and the Innovative Medicines Initiative (IMI), have, in recent years, strongly promoted and supported PI activities in research. A scoping review in 2020 (8) suggested that the number of published studies reporting PI activities was increasing, with PI taking place at different stages of the research process and with different methods being applied. A gap analysis carried out by the IMI-funded “Patients active in research and dialogues for an improved generation of medicines” (PARADIGM) project also identified several PI activities involving different groups of patients in the process of developing drugs and treatments.

However, the evaluation and reporting of the impact of PI still represent an important gap (8). The PARADIGM gap analysis work came to similar conclusions and highlighted the lack of publicly available information about the PI activities carried out in this context. When reporting exists, it is often fragmented and lacking the necessary details to make it possible for others to fully understand what was done, with whom, when, how, the outcomes—both positive and negative, the learning experiences, and the resulting value of the activity itself (PARADIGM tool “Guidance for Reporting and Dissemination of Patient Engagement Activities”).

Similarly, in their well-known GRIPP (Guidance for Reporting Involvement of Patients and the Public) guidelines, aimed at improving the quality and consistency of PI work and reporting, Staniszewska et al. (9) criticized the quality of reporting within scientific and peer-reviewed articles. They described reporting on PI as often being inconsistent and thus limiting the possibilities to learn from these research studies, and emphasized the importance of reporting what members of the public consider important to report.

In 2021, as part of activities carried out under an operating grant by the EU health programme, AE set out to identify 20 different examples of PI activities and methods used within the scope of European research projects in the field of neurodegeneration. As a first stage, we searched four key repositories/databases of European research into neurodegenerative disorders, namely CORDIS Community Research and Development Information Service, the JPND Research Database, IMI Project factsheets, and the Active and Assisted Living (AAL) programme website. After this, when necessary, we contacted researchers involved in the projects who were responsible for the PI work (when details were available) to ask for information. Finally, we hand-searched project websites for further information.

The first challenge was to identify projects that were planning or conducting PI activities and who was in charge of such activities. The databases and research platforms that we looked at contained a wealth of information but did not have specific search categories for PI work or any other information that could indicate that PI activities had been planned or conducted. The fact that different terminology is used to refer to PI, as stated earlier, maybe another relevant factor hindering the visibility and “searchability” of PI activities in this context. Some research funding bodies, for example, use the term PPI whereas others use the term PE (but to refer to what would be considered as PI under certain other classifications).

A second challenge was that there was very little information, if any, in the public domain about the nature of the PI work undertaken and of specific challenges linked to conducting PI with this population. It is possible that some projects had reported on the PI work in more detail but such reports may have been internal or were simply not readily available. Information on PI work was even more difficult to obtain if the project had already finished or the person responsible for the PI work had moved to a different position.

The PI work that we were able to identify varied considerably in terms of its approach, scale, impact on the project, the involvement of research partners, and how it had been reported. It was very difficult to capture differences or come to conclusions in relation to the models and methods used given the scarcity of the information available in the public domain. A couple of projects published peer-reviewed articles about some of the PI activities that had been conducted. Apart from this, the most common place where PI work was made public was the project website. In most of the IMI projects that we looked at, the project website had a dedicated section for PI or information for patients or the general public. However, the work carried out was not always described in sufficient detail, and, in particular, the information about how the PI input had been used and its impact on the project were even less likely to be in the public domain. This is an important gap, which makes it difficult to help ensure that the input provided by people living with dementia is used in a meaningful way (which has ethical, financial, and scientific implications). It is also important that people involved in the PI activities receive information about how their input was used (or why not) and what the impact of this was for the project.

## What are we calling for?

We very much welcome the commitment of European funding organizations to PI work in the field of dementia. We firmly believe that this is hugely beneficial to dementia research and it respects the right of people with dementia to have a voice in matters affecting their lives. The mapping exercise that we conducted in 2021 demonstrates the importance of ensuring that PI work is not only properly conducted but also properly documented.

There are often concerns about whether PI work is meaningful or a mere box-ticking exercise to obtain funding or ethics approval. To ensure that PI work truly contributes toward good research and that it is meaningful and well-conducted, it must be reported thoroughly and accurately. It cannot be a “black box” activity (e.g., “we conducted PI work”). National and European dementia research funders must insist not only on projects doing PI work but also on the deliverables and publicly available information about what was done. Amongst other things, further visibility of this work could be an inspiration for other organizations willing to conduct PI and could help to better understand the impact and benefits of PI in research projects.

We, therefore, recommend that:

• organisations' funding research should require at least one public deliverable for PI work and encourage researchers to publish details of the PI work on project websites or other places where the information is publicly accessible.
• academic publishers should require researchers to provide precise details of the nature, specific challenges, and impact of PI work in their manuscripts submitted for publication, and if PI was not carried out then to explain why this was the case.

## Data availability statement

The original contributions presented in the study are included in the article/supplementary material, further inquiries can be directed to the corresponding author.

## Author contributions

DG and AD-P conducted the mapping review. JG, DG, and AD-P drafted the article. JG, DG, AD-P, DL, and SM-B provided critical comments on the manuscript draft and approved the final version of the manuscript. All authors contributed to the article and approved the submitted version.

## Funding

The mapping review received funding under an operating grant from the European Union's Health Programme (2014–2020). This publication received funding from the Innovative Medicines Initiative 2 Joint Undertaking (JU) Under Grant Agreement No. 821513 (NEURONET). The JU receives support from the European Union's Horizon 2020 research and innovation programme and EFPIA and Parkinson's UK.

## Conflict of interest

The authors declare that the research was conducted in the absence of any commercial or financial relationships that could be construed as a potential conflict of interest.

## Publisher's note

All claims expressed in this article are solely those of the authors and do not necessarily represent those of their affiliated organizations, or those of the publisher, the editors and the reviewers. Any product that may be evaluated in this article, or claim that may be made by its manufacturer, is not guaranteed or endorsed by the publisher.

## References

[1] Ives J Damery S Redwod S. PI, paradoxes and Plato: Who's sailing the ship? Table 1. J Med Ethics. (2013) 39:181–5. 10.1136/medethics-2011-100150 22267385

[2] Gradinger F Britten N Wyatt K Froggatt K Gibson A Jacoby A. Values associated with public involvement in health and social care research : a narrative review. Health Expect. (2015) 18:661–75. 10.1111/hex.12158 24325553 PMC5060838

[3] Tritter JQ McCallum A. The snakes and ladders of user involvement: Moving beyond Arnstein. Health Policy. (2006) 76:156–68. 10.1016/j.healthpol.2005.05.008 16006004

[4] Gove D Georges J Rauf M Broeke J Jongsma K Claeys A. Overcoming Ethical Challenges Affecting the Involvement of People With Dementia in Research - Recognising Diversity and Promoting Inclusive Research. Luxembourg: Alzheimer Europe (2019).

[5] Owens AP Hinds C Manyakov NV Stavropoulos TG Lavelle G Gove D. Selecting remote measurement technologies to optimize assessment of function in early Alzheimer's disease: a case study. Front Psychiatry. (2020) 11:582207. 10.3389/fpsyt.2020.582207 33250792 PMC7674649

[6] Diaz A Gove D Nelson M Smith M Tochel C Bintener C. Conducting public involvement in dementia research: the contribution of the European Working Group of People with Dementia to the ROADMAP project. Health Expect. (2021) 13246. 10.1111/hex.13246 33822448 PMC8235878

[7] Stavropoulos T Lazarou I Diaz A Gove D Manyakov N Merlo Pich E. Wearable devices for assessing function in Alzheimer's disease: A European public involvement activity about the features and preferences of patients and caregivers. Front Aging Neurosci. (2021) 13:643135. 10.3389/fnagi.2021.643135 33912025 PMC8072390

[8] Miah J Parsons S Lovell K Starling B Leroi I Dawes P. Impact of involving people with dementia and their care partners in research: a qualitative study. BMJ Open. (2020) 10:1–12. 10.1136/bmjopen-2020-039321 33109666 PMC7592301

[9] Staniszewska S Brett J Simera I Seers K Mockford C Goodlad S. GRIPP2 reporting checklists : tools to improve reporting of patient and public involvement in research. BMJ. (2017) 358:j3453. 10.1136/bmj.j3453 28768629 PMC5539518

